# A cell‐type‐resolved meta‐analysis reveals glial DNA methylation changes associated with aging and Alzheimer's disease

**DOI:** 10.1002/alz.71864

**Published:** 2026-09-24

**Authors:** Uchit Bhaskar, Mark Z. Kos, Melanie A. Carless

**Affiliations:** ^1^ Department of Neuroscience, Developmental and Regenerative Biology The University of Texas at San Antonio San Antonio Texas USA; ^2^ Brain Health Consortium The University of Texas at San Antonio San Antonio Texas USA; ^3^ Division of Human Genetics, South Texas Diabetes and Obesity Institute School of Medicine University of Texas Rio Grande Valley Brownsville Texas USA

**Keywords:** aging, Alzheimer's disease, deconvolution, DNA methylation, epigenome‐wide association study, EWAS, glia

## Abstract

**INTRODUCTION:**

Epigenome‐wide association studies implicate DNA methylation in Alzheimer's disease (AD) pathology. Although recent studies identified epigenetic signatures within non‐neuronal cell types in disease risk, the role of the methylome in glial cell types (i.e., astrocytes, oligodendrocytes) in biological aging and AD pathogenesis is unclear.

**METHODS:**

In this study, we examined archived DNA methylation data across 13 cohorts and performed cell type deconvolution in silico to identify novel epigenetic signatures associated with aging and AD in glial cells.

**RESULTS:**

We observed pronounced age‐associated methylation signatures in astrocytes within the prefrontal cortex (PFC) and AD‐associated methylation signatures in oligodendrocytes of the entorhinal cortex. Astrocytes and neurons within the PFC emerge as key players in Braak stage‐associated methylation signatures. In oligodendrocytes, we observed amplification of age‐related effects of DNA methylation signatures with AD.

**DISCUSSION:**

Our study expands on previous findings and reveals glial‐specific methylation patterns associated with epigenetic aging and AD.

## Highlights

- Cell‐type deconvolution methods can be used to delineate cell‐specific epigenome‐wide associations across multiple cohorts.
- Astrocytes of the PFC exhibit substantial differential methylation with age.
- Oligodendrocytes in the EC show a high degree of differential DNA methylation with AD status.
- Both astrocyte and neuronal DNA methylation are highly concordant with the effect sizes (ESs) of tissue‐level Braak‐stage associations in the PFC.
- Age‐associated sites exhibit different methylation trajectories with AD, depending on the cell type.

## 1 BACKGROUND

Alzheimer's disease (AD) is largely characterized by the pathological accumulation of amyloid and tau proteins, leading to neuronal loss and dysfunction, which manifests as progressive cognitive decline. Recently, attempts have been made to unravel the role of glial cell types (including astrocytes and oligodendrocytes) in disease progression, including dysfunctions in maintaining central nervous system (CNS) homeostasis, blood–brain barrier integrity, myelination and axonal support, metabolism, and synapse maintenance.^1^, ^2^, ^3^ Several epigenome‐wide association studies (EWASs) have pointed to a role for DNA methylation in the etiology of AD and show differential DNA methylation across several CpG sites in relation to pathogenesis.^4^, ^5^, ^6^, ^7^, ^8^, ^9^, ^10^, ^11^, ^12^, ^13^, ^14^, ^15^, ^16^ Recent studies showed that among differentially methylated positions (DMPs) associated with Braak stages, a large fraction seemed to stem from non‐neuronal (NeuN^−^) cell types^11^ and underscored differences in neuronal and non‐neuronal CpG methylation in relation to AD,^5^ highlighting a critical need to resolve cell‐specific epigenetic contributions to disease states.

To date, most studies profiling DNA methylation in AD utilize bulk brain tissues, rather than single‐cell profiling, especially for glial subtypes. An alternative approach to assess cell‐type‐specific epigenetic contributions is to use computational deconvolution methods.^17^, ^18^, ^19^ One recent study used tensor‐composition‐based deconvolution analysis and identified a novel promoter hypomethylation signature at the *PEN‐2* gene, associated with amyloid plaque burden in neurons.^20^ However, further exploration is necessary to define changes in the glial epigenome in relation to both age and disease pathology.

In this study, we examined archived DNA methylation data from 13 different cohorts, totaling ∼3812 samples and spanning several different brain regions, to identify cell‐type‐specific methylomic variation associated with age and AD. We show that a significant proportion of DMPs associated with age occur in glial cell types (i.e., astrocytes and oligodendrocytes), as well as endothelial cells. AD associations tend to be highly specific to cell type and brain region. We observed a significant proportion of DMPs associated with AD status in oligodendrocytes compared to other cell types, particularly in regions affected early in the disease state (e.g., entorhinal cortex [EC]). DMPs within the prefrontal cortex (PFC), previously identified as being associated with Braak stage, are preferentially enriched in both astrocytes and neurons. Importantly, age‐associated DNA methylation signatures in oligodendrocytes across all cortical regions tend to be exacerbated with AD pathology, whereas age‐associated signatures of astrocytes seem to be decoupled with disease state. Together, this suggests the existence of distinct cell‐specific epigenetic programs in both healthy aging and neurodegeneration.

## 2 METHODS

### 2.1 Cohorts

**FIGURE 1 alz71864-fig-0001:**
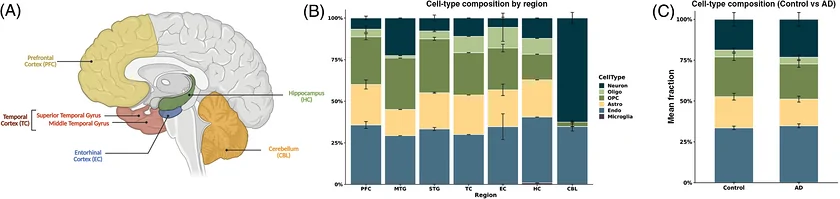
Overview of brain regions and cell types used in the study. (A) Representative schematic of human brain, highlighting brain regions analyzed in this study. Cell‐type composition estimates by (B) region and (C) disease status. Stacked bar sizes represent mean fractions across all cohorts analyzed. Error bars represent standard errors.

We identified a list of 13 archived brain‐specific AD EWASs, consisting of a total of 3812 independent samples (Table 1). All studies comprised at least 30 donors and provided publicly available data. We only included brain regions where we had data from at least two cohorts, each with a minimum of 30 individuals, and a total of at least 100 samples. The per‐cohort minimum ensures stable within‐cohort cell composition and covariate estimation, while the total‐sample threshold helps mitigate the reduced power that is typical of interaction‐based, cell‐specific association testing relative to conventional bulk EWAS models. Ten of the cohorts had DNA methylation data from 450K arrays, and the remaining three cohorts used EPIC arrays; we harmonized the probe set to assess the same CpG sites across all studies as previously described.^21^, ^22^ Across these studies, a total of seven brain regions were assessed – PFC, EC, middle temporal gyrus (MTG), superior temporal gyrus (STG), broad temporal cortex (TC), hippocampus (HC), and the cerebellum (CBL) (Figure 1A).

**RESEARCH IN CONTEXT**

1. **Systematic review**: We searched standard web‐based sources, including PubMed, Web of Science, and Google Scholar from inception to January 1, 2026, with no restrictions, using keywords such as “EWAS,” “DNA methylation,” “epigenome,” “aging,” “Alzheimer's disease,” “Braak,” “brain,” and “glia” in different combinations. Several large‐scale EWASs were previously undertaken at the brain‐tissue level to delineate epigenome‐wide associations in aging and AD, and these studies are appropriately cited. Our study utilized data from such archived studies to perform a large‐scale meta‐analysis using a cell‐type deconvolution framework to uncover glia‐specific epigenetic patterns.
2. **Interpretation**: From 13 cohorts, spanning ∼3812 samples, we identified cell‐type‐specific associations with age, AD status, and Braak stages across multiple cortical regions. We show the existence of distinct glia‐specific programs associated with both normative aging (e.g., PFC astrocytes are the most affected) and AD status (EC oligodendrocytes are the most differentially methylated). We also show that CpG sites identified at the tissue level to be associated with Braak stage are highly represented by astrocytes and neurons, but not other cell types. Lastly, our data also show distinct glia‐specific age‐to‐disease epigenetic trajectories, where age‐associated sites in oligodendrocytes have amplified effects in AD, but not in astrocytes. Together, our data highlight the importance of evaluating cell‐specific patterns of DNA methylation with disease‐related traits, which would allow a better understanding of disease etiology and cell‐type contributions.
3. **Future directions**: Our study uses a computational approach to identify cell‐specific epigenetic patterns. Future studies should validate the findings from this method and explore DNA methylation of individual cell types using either nuclear sorting methods, in vitro models, or single‐nucleus sequencing techniques. Longitudinal studies and/or cohorts with wider age‐distribution data are also required to assess the true age association, as well as to better assess age‐to‐disease trajectories. Coupling these data with other sequencing modalities, including chromatin, histone, transcriptomic, and metabolomic data, may help clarify the role played by DNA methylation in the disease context and help evaluate the potential utility of omics‐level approaches as biomarkers for AD diagnosis.

**TABLE 1 alz71864-tbl-0001:** Demographic details for all cohorts used in the meta‐analyses.

Accession ID	Study	Brain regions analyzed	Number of samples	Disease status		Sex (M/F)	Age in years (mean ± SD)	Ethnicity
GSE134379	Brokaw et al.^16^	Middle temporal gyrus	404	Control	179	103/76	84.18 ± 7.68	n.r.
				AD	225	101/124	83.28 ± 7.99	n.r.
		Cerebellum	404	Control	179	103/76	84.18 ± 7.68	n.r.
				AD	225	101/124	83.28 ± 7.99	n.r.
GSE66351	Gasparoni et al.^5^	Prefrontal cortex	63	Control	26	16/10	64.88 ± 16.69	n.r.
				AD	37	14/23	80.16 ± 8.36	n.r.
		Temporal cortex	65	Control	26	16/10	64.88 ± 16.69	n.r.
				AD	39	15/24	80.36 ± 8.19	n.r.
GSE72778	Horvath et al.^23^	Cerebellum	32	Control	9	5/4	62.89 ± 25.79	n.r.
				AD	23	6/17	91.13 ± 19.36	n.r.
		Hippocampus	25	Control	7	3/4	66.57 ± 20.18	n.r.
				AD	18	5/13	92.11 ± 20.16	n.r.
		Temporal cortex	29	Control	6	2/4	50.5 ± 22.43	n.r.
				AD	23	6/17	90.26 ± 20.08	n.r.
		Prefrontal cortex	41	Control	20	14/6	57.45 ± 20.15	n.r.
				AD	21	5/16	90.24 ± 19.98	n.r.
GSE109627	Lardenoije et al.^6^	Middle temporal gyrus	82	Control	36	17/19	84.67 ± 5.56	n.r.
				AD	46	22/24	85.07 ± 6.18	n.r.
GSE284764	Laroche et al.^9^	Prefrontal cortex	242	Control	111	57/54	83.45 ± 9.13	n.r.
				AD	131	58/73	81.82 ± 7.25	n.r.
GSE59685	Lunnon et al.^8^	Cerebellum	83	Control	23	13/10	76 ± 13.56	n.r.
				AD	60	21/39	86.15 ± 7.38	n.r.
		Entorhinal cortex	79	Control	21	12/9	79.62 ± 10.34	n.r.
				AD	58	19/39	86.5 ± 7.64	n.r.
		Prefrontal cortex	84	Control	24	12/12	78.62 ± 11.07	n.r.
				AD	60	21/39	86.4 ± 7.53	n.r.
		Superior temporal gyrus	87	Control	26	13/13	76.73 ± 13.16	n.r.
				AD	61	21/40	86.34 ± 7.48	n.r.
GSE105109	Smith A.R. et al.^12^	Cerebellum	96	Control	28	14/14	78.71 ± 11.14	n.r.
				AD	68	40/28	82.4 ± 8.3	n.r.
		Entorhinal cortex	96	Control	28	14/14	78.71 ± 11.14	n.r.
				AD	68	40/28	82.4 ± 8.3	n.r.
syn34166276	PITT ADRC	Prefrontal cortex	223	Control	28	19/9	76.14 ± 10.77	White: 25; Unknown: 3
				AD	195	92/103	79.87 ± 8.92	White: 194; Asian: 1
syn7357283	De Jager et al.^4^ (ROSMAP)	Prefrontal Cortex	546	Control	235	88/147	84.47 ± 5.45	White: 230; African American: 5
				AD	311	104/207	87.81 ± 3.54	White: 304; African American: 7
syn23633756	Thrush et al.^24^ (RR_APOE4)	Cerebellum	254	Control	80	26/54	85.95 ± 4.98	White: 75; African American: 5
				AD	174	59/115	88.06 ± 3.04	White: 169; African American: 5
		Prefrontal cortex	254	Control	79	26/53	85.9 ± 4.99	White:74; African American:5
				AD	175	59/116	88.07 ± 3.04	White: 170; African American: 5
GSE125895	Semick et al.^10^	Cerebellum	67	Control	43	24/19	60.63 ± 7.04	White: 17; African American: 26
				AD	24	11/13	79.47 ± 10.02	White:21; African American: 3
		Entorhinal cortex	69	Control	49	29/20	61.62 ± 7.61	White: 20, African American: 29
				AD	20	9/11	79.69 ± 9.64	White: 17; African American: 3
		Hippocampus	65	Control	48	29/19	61.71 ± 7.66	White: 19; African American: 29
				AD	17	8/9	81.54 ± 9.16	White: 14; African American: 3
		Prefrontal cortex	68	Control	47	28/19	61.54 ± 7.71	White: 18; African American: 29
				AD	21	11/10	79.95 ± 9.46	White: 18; African American: 3
GSE76105	Watson et al.^15^	Superior temporal gyrus	68	Control	34	16/18	80.56 ± 8.08	White: 23; Asian: 1; Hispanic: 5; African American: 5
				AD	34	17/17	78.94 ± 7.48	White: 29; African American: 1; Hispanic: 3; Unknown: 1
GSE80970	Smith R.G. et al.^13^	Prefrontal cortex	142	Control	68	34/34	82.76 ± 7.77	n.r.
				AD	74	20/54	88.53 ± 6.67	n.r.
		Superior temporal gyrus	144	Control	70	34/36	83.21 ± 8.13	n.r.
				AD	74	20/54	88.41 ± 6.76	n.r.

Abbreviation: n.r., not reported.

The study by Brokaw and colleagues^16^ consisted of 404 unique samples for two brain regions, the MTG and CBL. Gasparoni et al. had data available for 63 and 65 individuals for the PFC and TC, respectively.^5^ In one study by Horvath and others, data were available primarily for Huntington's disease but also included some individuals with AD that were included in our study; this comprised 32 samples from the CBL and 41 samples from the PFC.^23^ Although this study had assessed the HC and TC, we did not use these data in our final meta‐analysis design since it did not meet our criteria for having at least 30 samples. Lardenoije et al. had data available for the MTG in 82 individuals,^6^ and Laroche et al. had 242 samples from the PFC, conducted on an EPIC array.^7^ Several of our cohorts came from archived studies in the Medical Research Council London Neurodegenerative Disease Brain Bank, including one study published by Lunnon et al. containing data for the CBL, EC, PFC, and STG,^8^ as well as one by Smith and colleagues containing CBL and EC data.^12^ We also leveraged archived cohorts in the AD Knowledge Portal and assessed 546 samples in the Rush University Medical Center: Religious Order Study (ROS) and the Memory and Aging Project (MAP), i.e., ROSMAP cohort, for age and AD associations, as well as 734 samples for Braak stage associations in the PFC (all 450K array data).^4^ AD associations did not include samples that fell under the mild cognitive impairment category, but since Braak staging is a continuous measure, we included these in our later analyses. Additional samples from the ROSMAP cohort was also used in a second study (RR_APOE4) to assess 254 CBL and PFC samples using EPIC arrays. ^24^ We also obtained EPIC array data of 223 individuals for the PFC from the University of Pittsburgh Alzheimer's Disease Research Center from the AD Knowledge Portal. Semick and colleagues had available data across four brain regions (PFC, EC, CBL, HC), each comprising at least 60 individuals, that were used in this study.^10^ We also included one cohort consisting of 68 STG samples^15^ and another that contained 144 STG and 142 PFC samples,^13^ both previously published. Since MTG and STG broadly form a part of the overall temporal cortex, we grouped these as TC to increase power for the meta‐analysis. In total, we were able to assess 1626 samples in the PFC (nine cohorts), 794 samples in the TC (three cohorts each for MTG/STG, and one for TC), 154 samples in the EC (three cohorts), and 832 samples in the CBL (six cohorts), each for at least three to four cell types. Some regions were excluded because they did not meet our minimum sample‐size thresholds, including the HC, which had a sample size too small to be used for any of our analyses, and the EC, which was excluded from the Braak stage analyses; both regions are believed to play key roles in AD progression.[Table alz71864-tbl-0001], [Fig alz71864-fig-0001]

### 2.2 Data preprocessing

All computational analyses were carried out in R version 4.5.1. IDAT files from raw Illumina HumanMethylation 450K or EPIC arrays for each cohort were imported, and an *RGChannelSet* object was created using the *minfi* package (version 1.56.0).^25^ Sample‐level quality control (QC) was performed using an approach similar to that used in a previous publication.^14^ Samples were removed if they had (1) bisulfite conversion efficiency < 80%, (2) abnormal background intensity of negative probes (<1000), (3) outlier mean methylated or unmethylated intensities (>3 SDs), (4) discordance between predicted and reported sex, or (5) genetic identity mismatches detected using single‐nucleotide polymorphism (SNP) probes where available. Probe‐level QC was carried out using the *minfi* and *ewastools* (version 1.8.0) packages^26^ and implemented using the ChAMP package (version 2.21.1) in R.^27^ Probes present on sex chromosomes, those containing SNPs at the CpG interrogation site, and those with known cross‐reactivity were removed. Background correction and dye‐bias normalization were carried out using the NOOB method, implemented in *minfi*. Probes with detection *p* values > 0.01 and those with less than three beadcounts in at least 5% of the samples were removed. Signal intensities of probes passing quality metrics were converted to beta values. In cohorts where raw IDAT files were not available, signal intensity data files were used, and a *MethylSet* object was generated using *minfi*. All other probe‐level QC was performed as before, except for beadcount filtering, for which data were not available. Finally, BMIQ‐based normalization^28^ was implemented on beta value matrices using the ChAMP package.

### 2.3 Cell‐type deconvolution and differential methylation analysis

To estimate cell proportions within each cohort, we used a previously published DNA methylation reference matrix (EpiSCORE) for brain tissues^29^ that was implemented in a prior deconvolution study.^20^ Bulk data were processed for each brain region separately. First, the constAvBetaTSS function from the EpiSCORE package (version 0.9.6) was used to map CpG loci to Entrez IDs to derive promoter‐level methylation estimates. Cell proportions were then estimated using a weighted robust partial correlation method, implemented in EpiSCORE. The reference matrix in EpiSCORE derives tissue‐specific DNA methylation by translating cell‐type marker information from single‐cell RNA sequencing (RNA‐seq) reference atlases into predicted methylation profiles at regulatory regions. It can be used to estimate proportions for at least six unique brain cell types.^20^ Because we detected <0.5% of microglia across several cohorts, these were dropped from further analysis. Oligodendrocyte estimates were relatively small, so we grouped these with oligodendrocyte precursor cells (OPCs) for further analyses, as was done previously,^20^ to allow better power for the detection of significant associations. This common “Oligo_OPC” pool is hereafter referred to as oligodendrocytes for simplicity.

Cell‐type‐specific association analysis for age, AD, and Braak stage was performed using the CellDMC framework, implemented in the EPIDISH package (version 2.28.0).^30^, ^31^ This method models interactions between estimated cell type proportions and phenotypic variables to identify CpGs exhibiting differential methylation effects attributable to a given cell type. To do this, we first harmonized CpG probes to those existing on both the 450K and EPIC arrays, then prespecified association models for testing. Briefly, for age, AD status, and Braak stage, we performed region‐stratified analysis with criteria for a minimum sample size of 30 per cohort, with at least five individuals contributing to each phenotype outcome group. These criteria were imposed to avoid rank‐deficient models and unstable estimation of CellDMC interaction effects, which can occur when phenotype or region groups contain very small sample numbers. For age associations, we controlled for sex and diagnosis as covariates and modeled age as a continuous variable. For AD, we used age and sex as covariates, modeling AD diagnosis as a binary variable and Braak stage as a continuous variable. To control for unmeasured technical and biological confounders, we used surrogate variables (SVs), implemented using the *sva* package (version 3.58.0).^32^ Surrogate variable analysis (SVA) models included the outcome of interest (age, AD, or Braak stage), covariates, and cell‐type fractions. The number of SVs was selected adaptively by iteratively increasing the number of SVs until the genomic inflation factor (λ) for the given outcome term per region was reduced to ≤1.2, as previously implemented by Smith et al.^14^ If baseline inflation was already below the threshold, no SVs were included for that model. Finally, CellDMC models were fit using beta values as the response variable, with phenotype terms, covariates, and selected SVs included as predictors. Cell‐type‐specific effect sizes (ESs), standard errors, and *p* values were extracted for each CpG, and multiple testing correction was applied using both the Benjamini‐Hochberg false discovery rate (FDR) method^33^ and Bonferroni correction.^34^ We mainly report FDR‐based significance metrics in this study, although Bonferroni adjustments are available upon request. Unless otherwise specified, FDR < 0.05 was used as a statistical significance threshold for all analyses. For each model and cell type, genomic inflation was computed after association tests, and quantile‐quantile plots were generated to assess residual test statistic inflation, and association results eligible for meta‐analysis were exported for further processing.

### 2.4 Region‐stratified cell‐specific meta‐analysis

Cell‐type‐specific association results from individual cohorts were meta‐analyzed separately for age, AD status, and Braak stage, using a fixed‐effects inverse variance weighting (IVW) framework. Meta‐analysis was performed at the level of CpG × region × cell type by integrating ESs and SEs derived from the CellDMC output. CpG‐level results were required to be supported by at least two independent cohorts to be eligible for meta‐analysis. To control for residual test statistic inflation and bias at the cohort‐region‐cell‐type level, ESs were corrected using the BACON method before meta‐analysis.^35^ Groups of phenotype × region × cell type were excluded from further analysis if genomic inflation exceeded 2.0 before BACON correction or remained >1.5 after correction, ensuring that only well‐calibrated cohorts contributed to the meta‐analysis. We implemented the inverse variance‐weighted fixed‐effects model via the *metagen* function within the meta package (version 8.2.1).^36^ Random‐effects models were not included for primary inference due to the typically small number of contributing cohorts; however, heterogeneity statistics (Cochran's Q, *I* ^2^) are reported. FDR significance was set at 0.05.

### 2.5 Differentially methylated region (DMR) analysis

DMRs were identified from cell‐type‐specific meta‐analysis results using the *comb‐p* method^37^ implemented in Python (version 2.7), as previously described.^14^ Regions were spatially aggregated within a 500‐bp window and seeded using a nominal CpG *p* value threshold of 0.05, then corrected for multiple testing using the Sidak method. DMRs were annotated to nearby genes and genomic features (*hg19*), and representative loci were visualized using genomic context tracks, implemented using the *Gviz* package (version 1.54.0) in R.^38^

### 2.6 ES rank enrichment analysis

To evaluate cell‐type contributors to tissue‐level Braak stage DMPs previously reported by Smith et al. (*hereafter* called Smith‐CpGs or Smith‐DMPs for simplicity),^14^ we used a rank‐based enrichment analysis of ESs within each region and cell type. For each cell type per region, all ∼400,000 CpG sites were ranked by their absolute z‐score (defined as ES/SE) of the Braak association from the meta‐analysis. Smith‐CpGs were treated as a predefined reference set, and enrichment was assessed by comparing the distribution of their absolute z‐scores to that of all other CpGs using the Wilcoxon rank‐sum tests and permutation‐based area under the curve (AUC) statistics (1 × 10^4^ permutations). This approach assesses whether previously reported tissue‐level Braak‐associated CpGs tend to exhibit a stronger cell‐type‐specific Braak effect than expected by chance. Results were visualized using ridge density plots.

To further characterize the directional concordance between Smith‐CpGs and cell‐type‐specific Braak associations (PFC astrocytes and neurons), we performed leading‐edge concordance analysis. Smith‐CpGs were first ranked in order of decreasing absolute z‐scores, such that the CpGs with the strongest reported Braak associations were evaluated first. Directional concordance was then computed cumulatively across the ranked list. At each rank threshold *k*, concordance was defined as the proportion of top *k* CpGs whose ES had the same direction in the cell‐type‐specific meta‐analysis as previously reported by Smith et al. Concordance values therefore range from 0 to 1, with 0.5 representing random directional agreement. Cell‐type differences within the leading edge were evaluated using permutation‐based tests, comparing directional concordance over the top 50 CpGs (1000 permutations). Differences in directional concordance between the two cell types were interpreted qualitatively as reflecting differences in how strongly or broadly tissue‐level Braak‐associated CpGs aligned directionally with cell‐specific Braak effects across the ranked sites.

### 2.7 Cell‐specific age‐to‐disease trajectory analysis

Age‐to‐disease trajectories were assessed by comparing direction and effect sizes of CpGs nominally significantly associated with age (*p <* 0.05) between cell‐type‐specific age and AD associations. ES amplification was quantified as the slope of the regression between age and AD ES estimates for the age‐associated DMPs. Pearson correlation was also computed to test the extent to which the same CpGs contributed to both age and disease associations in a given cell type and region. Concordance indicates that the AD effects were in the same direction as the age effects, while ES scaling (regression) indicates whether AD‐associated changes are comparable to or exceed age‐associated changes.

### 2.8 Gene ontology (GO), pathway, and other visualizations

CpGs were annotated using the Illumina (UCSC) gene annotation database using the *minfi getAnnotation* function. Annotations are based on their proximity to known transcription start sites in the genome. Functional enrichment of CpGs identified in the meta‐analysis was performed using the *gometh* function, implemented in the *missMethyl* package (version 1.44.0) in R.^39^ All other data visualizations, including summary plots, Manhattan plots, and volcano plots, were generated using the *ggplot2* package (version 4.0.2) in R.

## 3 RESULTS

We analyzed cell‐type‐specific DNA methylation differences associated with age and AD status across a total of seven brain regions (PFC, MTG, STG, EC, TC, HC, and CBL). Since prior EWASs pointed to roles for non‐neuronal cell types in disease pathology, and age is the largest risk factor for AD, we were interested in evaluating regional glial cell vulnerability associated with both aging and AD. For our initial analysis, we focused on either age as a continuous variable or AD status as a binary variable, since stage‐specific disease data were not available across all cohorts. However, for additional analysis, we also used Braak stage as a continuous variable to assess cell‐specific associations when available. We employed a cell‐type decomposition method, using an existing EpiSCORE brain reference panel,^29^, ^40^ for each study and performed cohort‐wise, region‐stratified, cell‐specific association analyses using CellDMC for age, AD, and Braak stages.^30^ Although EpiSCORE has been validated as a strategy for deriving methylation reference matrices from single‐cell RNA‐seq atlases,^41^, ^42^ reference‐based estimates depend on cell types, regions, and cell states represented in the reference. It should be noted that the brain reference used in this study, while directly applicable to cortical tissue, may not fully capture region‐specific methylation signatures, as indicated by previously reported cell‐type‐specific estimation biases of the EpiSCORE algorithm.^43^, ^44^

Cell‐type compositions were largely similar across multiple cortical regions (Figure 1B; Table S1), irrespective of healthy and disease states (Figure 1C). Because these proportions are derived from reference‐based methylation deconvolution estimates, they should be interpreted as such, rather than as direct histological cell counts. Published single‐nucleus and deconvolution‐based studies of human cortex report substantial representation of neuronal, astrocytic, oligodendrocytic, microglial, endothelial, and perivascular populations. However, the exact proportions vary by brain region, sampling and nucleus isolation methods, disease status, and reference taxonomy. Here, we used the EpiSCORE‐derived estimates primarily to enable cell‐type‐specific association modeling rather than to infer absolute cellular abundance. Overall, across cortical and hippocampal tissues, neurons accounted for 9.53 ± 1.36% of total cells, astrocytes 21.41 ± 0.94%, endothelial cells 36.34 ± 1.66%, oligodendrocytes 8.03 ± 1.82%, OPCs 24.33 ± 2.09%, and microglia < 0.6% (0.35 ± 0.17%). In the CBL, we saw a distinct pattern, with only neurons (60.4 ± 2.53%), oligodendrocytes/OPCs (3.24 ± 0.79%), and endothelial cells (36.19 ± 1.95%) making up the cell types. It is possible that neuronal fractions may be underestimated because EpiSCORE's promoter‐level CpG‐only reference does not capture non‐CpG hypomethylation that distinguishes neurons at the single‐cell level, while endothelial fractions may be influenced by the retention of vascular tissue in bulk tissue dissection and the absence of a separately modeled pericyte/stromal compartment in the reference. Reassuringly, the means and variabilities of our cell‐type estimates across different brain regions are not correlated with DMP burden for AD or age (Figure S1), and these cell fraction estimates did not differ significantly by disease status (Figure 1C), reducing concern that systematic differences in estimated cell composition alone account for the observed disease associations.

### 3.1 Cell‐Type deconvolution uncovers unique glia‐specific age‐associated DMPs

**FIGURE 2:**
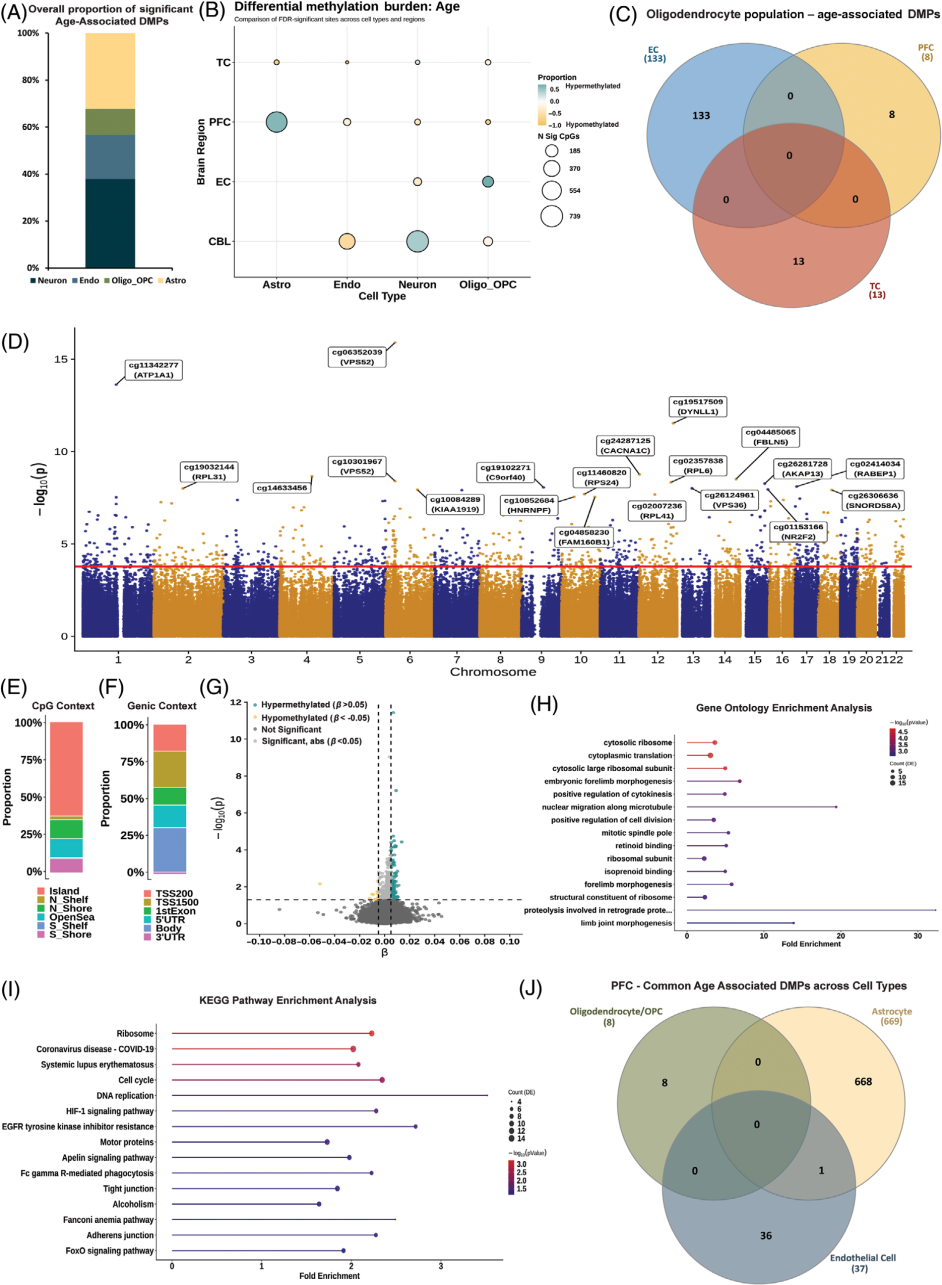
Region‐stratified cell‐type deconvolution‐based meta‐analysis identifies glia‐specific aging programs. (A) Stacked bar chart showing overall proportions of significant age‐associated differentially methylated positions (DMPs) across all regions. Significance set at FDR < 0.05. (B) Bubble plot showing differential methylation burden associated with age across all cell types × regions analyzed. Color scale represents proportion of sites seen to be hypermethylated (teal) and hypomethylated (orange). Bubble size indicates number of significant CpGs. Also see Figure S1 for Bonferroni corrected associations. (C) Venn diagram of age‐associated DMPs in oligodendrocyte fractions across all cortical regions, showing no shared loci. (D) Manhattan plot for meta‐analysis on prefrontal cortex astrocytes (*N =* 1626 individuals), with the 20 most significant DMPs annotated with either CpG ID or Illumina UCSC gene name or both. The *x*‐axis shows chromosomes 1 through 22, and the *y*‐axis shows −log_10_(*p*), with the horizontal red line denoting FDR significance (0.05). (E) Stacked bar chart showing proportion of significant age‐associated DMPs in PFC astrocytes that are enriched for different CpG contexts, based on Illumina UCSC annotations. (F) Stacked bar chart showing proportion of significant age‐associated DMPs in PFC astrocytes that are enriched for different genic positions, based on Illumina UCSC annotations. (G) Effect size volcano plot of age‐associated DMPs in PFC astrocytes. Horizontal line identifies FDR threshold of 0.05, whereas the vertical lines indicate effect size, *|β|* > 0.05. (H) Lollipop plot of 15 most enriched Gene Ontology terms for age‐associated DMPs in prefrontal cortex astrocytes, ordered by counts. The *x*‐axis represents fold enrichment and color scale represents −log_10_(*p*). (I) Lollipop plot of 15 most enriched Kyoto Encyclopedia of Genes and Genomes pathway analysis terms for age‐associated DMPs in PFC astrocytes, ordered by highest counts. The *x*‐axis represents fold enrichment and color scale represents −log_10_(*p*). (J) Venn diagram of common age‐associated DMPs across astrocytes, oligodendrocytes, and endothelial cells within the PFC.

Age is a significant risk factor for the development of several neurodegenerative disorders. Previously, epigenetic age acceleration was shown to be associated with AD‐related pathology, including neuritic plaques and neurofibrillary tangles,^45^ suggesting that age‐related methylation might be a key player in mediating neurodegenerative phenotypes. Importantly, identifying age‐associated methylation in a cell‐type‐specific manner will help us better understand cell‐type vulnerability patterns associated with late‐stage brain disorders. Since cell‐type deconvolution methods substantially reduce statistical power due to the high dimensionality of the data, we chose to correct for multiple testing and assess significance using a FDR (Benjamini‐Hochberg) threshold of 0.05. With this, we identified 2101 significant DMPs, of which neurons, astrocytes, oligo‐OPCs, and endothelial cells represented 37.93%, 32.27%, 11.09%, and 18.71% of total hits, respectively (Figure 2A). Neurons contributed to a large proportion (∼63%) of the significant age‐associated cerebellar DMPs; whereas in cortical tissues, the largest number of detectable age‐associated DMPs were observed in glial cell fractions (Figure 2B; Figure S2 shows data for Bonferroni corrected associations).

Astrocytes harbored 93% of the significant DMPs in the PFC (669 CpG sites) and 32% (nine DMPs) in the TC. Oligodendrocytes held 71.5% of the significant DMPs in the EC (133 CpG sites), 1.1% in the PFC (eight DMPs), and 46.4% in the TC (13 DMPs). We did not observe any intersection of the oligodendrocyte‐significant DMPs across the three cortical regions (Figure 2C), indicating brain region‐specific CpG methylation patterns with age among these cells. Of the 133 DMPs in the EC oligodendrocytes (Figure S3A), 113 are hypermethylated with age, and 20 sites are hypomethylated. Among the top 10 hits, we observed hypermethylation at a site within the *DCDC2* gene body (cg11563656, ES = 0.016764436, *p =* 3.93 × 10^−10^), previously identified as a predictor for episodic memory maintenance in aged adults.^46^ GO analysis showed enrichment for neurodevelopmental processes, including Notch signaling, and those involved in protein metabolism, to be associated with age in these cells (Figure S3B).

Since the PFC represented our largest sample set, and astrocytes showed the highest number of significant DMPs in this region, we focused on these cells. Of the 669 DMPs, 567 sites mapped to known genes (∼84.7%) in the genome, including novel associations at three sites within the *VPS52* gene (cg06352039, *p =* 1.24 × 10^−16^; cg09112371, *p =* 6.75 × 10^−6^; cg10301967, *p =* 3.94 × 10^−9^), as well as hits at genes with known astrocyte functions such as ionic and glutamate transport (*ATP1A1*, cg10442913, *p =* 2.95 × 10^−8^; cg13079047, *p =* 2.87 × 10^−7^), calcium signaling (*CACNA1C*, cg24287125, *p =* 1.73 × 10^−9^), and astrocyte reactivity (*FBLN5*, cg04485065, *p =* 3.06 × 10^−9^) (Figure 2D). We also observed other significant DMPs mapped to genes of the SLC, ADAM, and KLF families (Table S2) and a site mapped to the *PRDM2* gene (cg23813012, *p =* 8.54 × 10^−7^) that was previously observed in an age‐association EWAS of sorted neuronal and non‐neuronal cell types.^5^

Of the 669 identified significant DMPs, nearly 60% are located within CpG islands, about 25% in adjacent CpG “shore” regions (within 2 kb of islands), and the remainder in CpG “shelves” (2 to 4 kb) and “open sea” regions (>4 kb) (Figure 2E). Over a fourth of the sites map to known gene bodies, and nearly 40% of sites map to regions upstream of the transcription start site (most between 200 and 1500 bp upstream) (Figure 2F). A smaller fraction of the sites map to either 5ʹ UTRs (∼15%) or first exons (∼9%). Of the 669 significant DMPs, most showed small ESs, with only 13 hypomethylated sites having an ES < −0.005 and 114 hypermethylated sites having an ES > 0.005 (Figure 2G). The highest ES was observed for cg14633456 (ES = 0.0136, *p =* 2.16 × 10^−9^), a site previously implicated in aging.^47^ GO (Figure 2H) and KEGG pathway analysis (Figure 2I) showed significant enrichment (*p <* 0.05) for GO terms related to translation and protein processing (e.g., cytosolic ribosome, cytoplasmic translation), cell division (e.g., positive regulation of cytokinesis and cell division, cell cycle, DNA replication), and those involved in phagocytic and inflammatory processes, all of which are known to be disrupted with age. DMR analysis in the PFC‐astrocytes identified 224 significant regions (Sidak corrected *p <* 0.05), with the most significant DMR mapping to the *ATP1A1* gene (Sidak *p =* 1.66 × 10^−21^) (Table S3). Sixty‐five of the significant PFC‐astrocyte loci are also nominally significant in the TC (Figure S4A), with a significantly positive ES correlation (*r =* 0.78, *p =* 8.77 × 10^−15^; Figure S4B), suggesting potential shared mechanisms for age‐related changes across the cortex in astrocytes. Finally, we assessed whether any of the sites significantly associated with age in astrocytes were also implicated in other cell types in the PFC (Figure 2J). In the oligodendrocyte pool, only eight sites showed significant associations with age (FDR < 0.05), while 17 significant DMPs were found in neurons – none of which were common to those seen in astrocytes, and in endothelial cells only one of the 37 age‐associated sites was observed among the astrocyte DMPs.

### 3.2 Oligodendrocytes exhibit region‐specific differential methylation associated with lipid metabolism in AD

**FIGURE 3:**
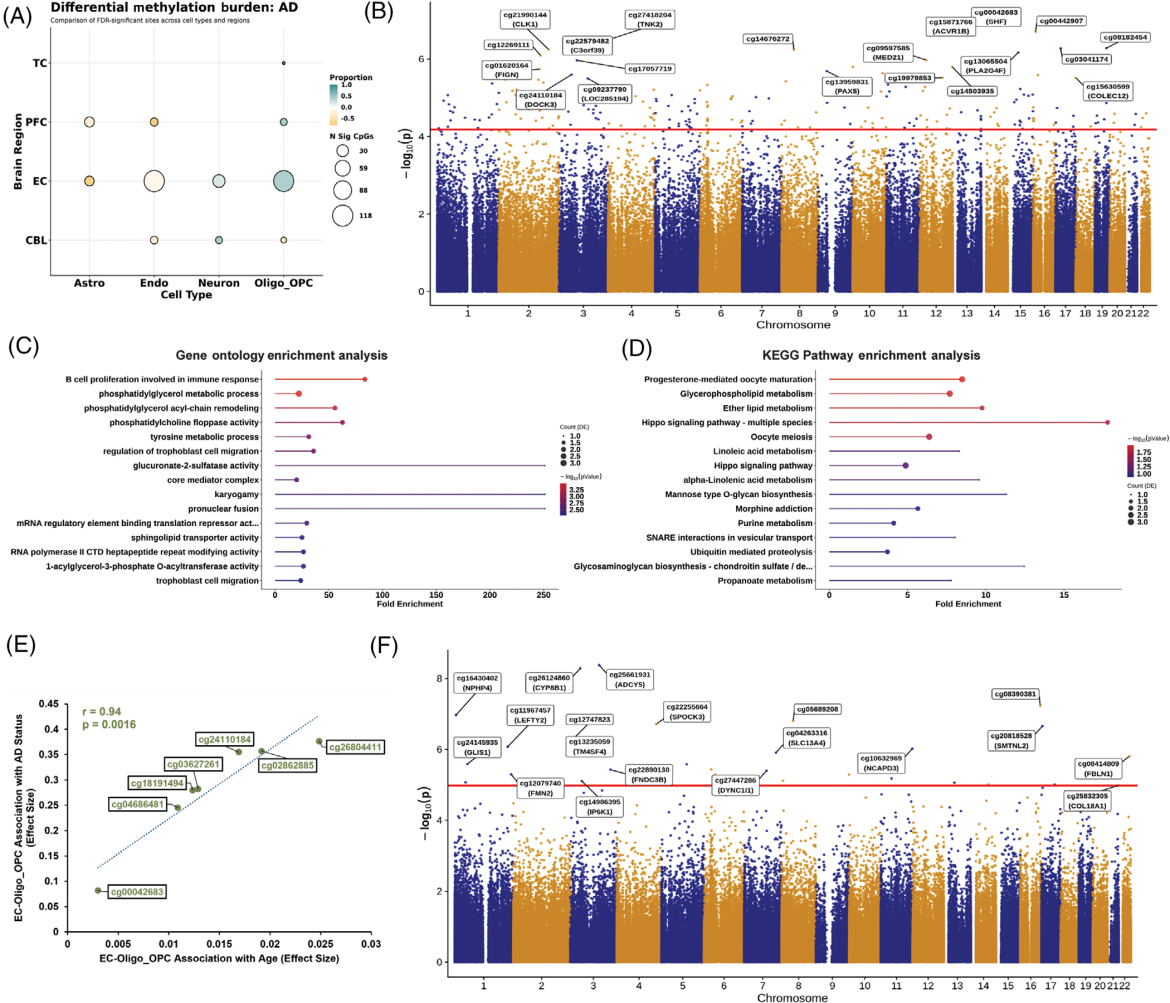
AD‐associated DMPs exhibit region‐ and cell type specificity. (A) Bubble plot showing differential methylation burden associated with AD status across all cell types × cortical regions analyzed. Color scale represents proportion of sites seen to be hypermethylated (teal) and hypomethylated (orange). Bubble sizes indicate number of significant CpGs. (B) Manhattan plot for meta‐analysis on entorhinal cortex oligodendrocytes (*N =* 154 individuals), with the 20 most significant DMPs annotated with either CpG ID or Illumina UCSC gene name or both. The *x*‐axis shows chromosomes 1 through 22, and the *y*‐axis shows −log_10_(*p*), with the horizontal red line denoting FDR significance (0.05). (C) Lollipop plot of 15 most enriched Gene Ontology terms for AD‐associated DMPs in EC oligodendrocytes, ordered by counts. The *x*‐axis represents fold‐enrichment and color scale represents −log_10_(*p*). (D) Lollipop plot of 15 most enriched Kyoto Encyclopedia of Genes and Genomes pathway analysis terms for AD‐associated DMPs in EC oligodendrocytes, ordered by counts. The *x*‐axis represents fold enrichment. Color scale represents −log_10_(*p*). (E) Correlation scatter plot of significant DMPs shared between age and AD associations in EC oligodendrocytes (*r =* 0.94, *p =* 0.0016, assessed by *t*‐test). (F) Manhattan plot for meta‐analysis on prefrontal cortex astrocytes (*N* = 1626 individuals), with the 19 most significant DMPs annotated with either CpG ID or Illumina UCSC gene name or both. The *x*‐axis shows chromosomes 1 through 22, and the *y*‐axis shows −log_10_(*p*), with the horizontal red line denoting false discovery rate significance (0.05).

We performed association analysis using AD status as a binary variable while controlling for age and sex in a region‐stratified manner. Like our age‐association study, we utilized a fixed‐effects IVW method to meta‐analyze these effects across the PFC, TC, EC, and CBL. Overall, in comparison to our age meta‐EWAS, we observe far fewer significant associations with AD status across all regions and cell types (20 in total) at a Bonferroni significance threshold of 0.05 (*p =* 1.25 × 10^−7^ for ∼400,000 sites) (Figure S5A). With a FDR threshold of 0.05, we observe only 36 significant hits across all cell types in the PFC, 20 hits in the CBL, and only one significant DMP in the TC (Figure 3A). However, AD association in the EC shows a significantly higher proportion of DMPs (283 significant sites across all cell types; Figure S5B), indicating region‐specific differences in DNA methylation associated with AD. As with age, glial cells and, to some extent, endothelial cells represented the highest proportion of detectable DMPs in the cortical regions.

EC oligodendrocytes exhibit a substantial proportion of differentially methylated sites associated with AD. Of these, 80 sites are hypermethylated with AD, while 34 sites are hypomethylated (Figure 3B and Figure S5B). Our most significant hit is for a CpG site located within the gene body of the *SHF* gene (cg00042683, ES = 0.082, *p =* 7.65 × 10^−8^), known to play a critical role in oligodendrocyte fate specification and found to be downregulated in AD.^48^ Critically, we see hypermethylation at a novel CpG site in the 3’ UTR of the *ABCA1* gene (cg13559409, ES = 0.24, *p =* 1.77 × 10^−5^), a key player of cholesterol transport, and lipidation of the well‐known late‐onset genetic risk factor, *APOE*.^49^, ^50^ Other notable significant AD‐related DMPs include *DOCK3* (a presenilin binding protein), *PDE4D* (calcium modulator impacting memory), and *SERPINI1* (important for limiting excitotoxicity) (Table S4). GO and KEGG pathway enrichment analysis for DMPs in the EC oligodendrocytes identified several terms relevant to fatty acid and lipid metabolism‐related processes, as well as those involved in proteoglycan biosynthesis (Figure 3C,D), supporting a role for DNA methylation in modulating dysregulation of functional oligodendroglial cell states. Of note, DMR analysis of these results shows significant hits at a neuroinflammatory gene, *C1QTNF7* (Sidak corrected *p =* 4.59 × 10^−7^), containing seven sites, as well as three sites within the *PLEC* gene (Sidak corrected *p =* 7.03 × 10^−5^), previously implicated as a critical player in pathological tau accumulation (Table S5).^51^ Our study now strengthens these findings by implicating a role for abnormal DNA methylation in these processes.

Since we observed several significant hits associated with age in EC‐oligodendrocytes, we asked whether any of these sites are shared between age and AD, and if so, how they are correlated. A total of seven CpG sites are significantly associated with both age and AD in EC oligodendrocytes (Figure 3E). While all seven DMPs are hypermethylated with both age and AD with a Pearson's correlation of 0.94 (*p =* 0.0016, Student's *t*‐test), effect sizes for individual sites are much higher in AD associations (in the range of 0.08 to 0.38) than in the age association (range: 0.003 to 0.02). These loci occupied multiple genes, which were previously implicated in AD‐related pathogenesis, and include our most significant AD hit within the *SHF* gene body (cg00042683), as well as sites within gene bodies of *DOCK3* and *PDE11A* (Table S6). The substantial increase in ES with AD at these sites points to disease‐specific exacerbation of age‐related methylation changes, likely specific to oligodendrocytes of the EC. Interestingly, to our knowledge, no previous EWAS of AD reported a significant association for these sites, thereby highlighting plausible novel candidates of AD‐related age exacerbation in oligodendrocytes. We observed no shared significant sites in the oligodendrocyte population from the other cortical regions (PFC and TC).

Within the PFC, similarly to the age‐associated methylation patterns, astrocytes showed the highest number of DMPs. Of 36 significant DMPs across all cell types, 19 sites are detected in astrocytes (Figure 3F), of which seven are hypermethylated and the remaining 12 are hypomethylated (Table S7). Our most significant DMP is at a site (cg25661931) within a CpG island in the first exon of the *ADCY5* gene (ES = −0.091, *p =* 4.16 × 10^−9^). *ADCY5* encodes a member of the adenylate cyclase family of cAMP producers and was previously linked to the accumulation of hyperphosphorylated tau in AD.^52^ Also, cg25661931 within *ADCY5* has been linked to an epigenetic switch point associated with aging in a recent study.^53^ Differential methylation at the *HOXA3* gene cluster has been associated with AD pathology in the PFC.^5^, ^13^, ^14^ In astrocytes, we identified a nominally significant DMP (ES = 0.12, *p =* 0.008) within this gene (cg19048532). Nevertheless, most of the DMPs identified in the PFC astrocytes showed only low ESs in association with AD, and only two sites were shared with age‐associated DMPs in PFC astrocytes. We also observed no significant shared DMPs between astrocytes in the EC and PFC with disease status (data not shown). However, DMR analysis identified 20 significant regions associated with AD in PFC astrocytes (Table S8). Of these, a significantly associated DMR is present within *SLC5A5* (Sidak corrected *p =* 0.001, seven CpG sites), an ion transport gene previously shown to be protective for AD and frontotemporal dementia.^54^

### 3.3 Astrocytes and neurons drive methylation signals associated with Braak stages

**FIGURE 4:**
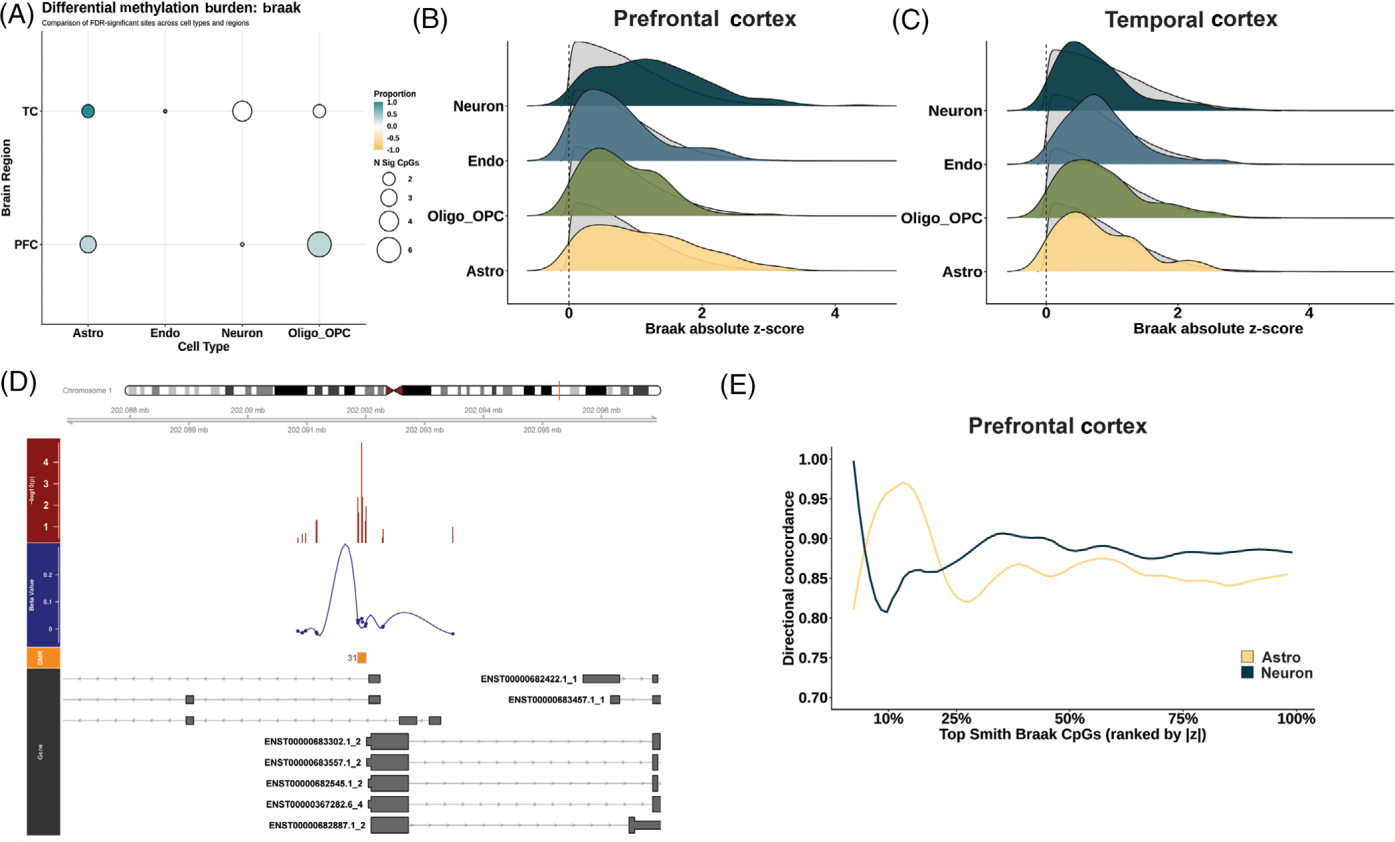
Astrocytes and neurons show distinct differential methylation trajectories in association with Braak stages. (A) Bubble plot showing differential methylation burden associated with Braak stage across all cell types × cortical regions analyzed. Color scale represents proportion of sites seen to be hypermethylated (teal) and hypomethylated (orange). Bubbles size indicates number of significant CpGs. (B) Representative ridge plots for rank‐based enrichment analysis of effect sizes for bulk tissue Braak‐associated CpGs, identified in Smith et al., ^14^ across all cell types in the prefrontal cortex. The *x*‐axis shows absolute z‐scores, defined as ES/SE for Braak associations of each cell type in the meta‐analysis. For each cell type, absolute z‐scores of all ∼400,000 CpG sites are ranked, and probability distributions are shown as gray shaded ridges. Two hundred thirty‐six Braak‐associated CpGs identified as significantly associated in bulk PFC by Smith et al. are treated as a predefined reference set, and absolute z‐score distributions of these sites are represented by colored ridges. (C) Representative ridge plots for rank‐based enrichment analysis of effect sizes for bulk tissue Braak‐associated CpGs, identified in Smith et al., ^14^ across all cell types in the temporal cortex. The *x*‐axis shows absolute z‐scores, defined as ES/SE for Braak associations of each cell type in the meta‐analysis. For each cell type, absolute z‐scores of all ∼400,000 CpG sites are ranked, and probability distributions are shown as gray shaded ridges. Eighty‐eight Braak‐associated CpGs identified as significantly associated in bulk PFC by Smith et al. are treated as a predefined reference set, and absolute z‐score distributions of these sites are represented by colored ridges. (D) Differentially methylated region (DMR) analysis identifies regions previously identified as Braak‐associated DMPs. Representative genome visualization plot of one such DMR at *GPR37L1* locus. The red track shows *p* value significance, and the blue track shows ES of CpGs at this DMR in the meta‐analysis. The orange track shows the DMR, and the genome tracks are shown below. (E) Leading‐edge directional concordance analysis of PFC astrocytes and neurons for all Braak‐associated CpGs identified at the tissue level by Smith et al. Smith‐CpGs are ranked in order of decreasing absolute z‐scores along the *x*‐axis. Directional concordance between tissue‐level ES and cell‐type‐specific ES is computed cumulatively across the ranked list and represented along the *y*‐axis. Concordance value of 0.5 represents random directional agreement.

Although Braak stage data were not publicly available for all cohorts in our analysis, we conducted a meta‐analysis for cohorts that had these data available. In total, we were able to assess Braak stage associations in the PFC for 1506 samples (across six cohorts) and in the TC (combined STG, MTG, and broad TC) for 296 samples (three cohorts) (Table S9). Of the three cohorts that had EC data available, only two provided Braak stage as a phenotype, and one of these did not meet model rank requirements, so we excluded the EC from our analysis. We observed a total of 10 significant DMPs in the PFC and nine in the TC at a FDR‐adjusted threshold of 0.05 (Figure 4A). Specifically, within the PFC, we observed three DMPs in astrocytes, six in the oligodendrocytes, and one in neurons, whereas in the TC, we saw two DMPs each in the astrocytes and oligodendrocytes, one in endothelial cells, and four in the neurons. There was no overlap of significant DMPs between AD (as a binary trait) and Braak staging association analyses (data not shown). DMR analysis indicated cell‐type‐specific methylation signatures in the PFC, implicating 32 significant (Sidak *p <* 0.05) astrocyte‐associated DMRs, 16 oligodendrocyte‐associated DMRs, 13 neuron‐associated DMRs, and two endothelial cell‐associated DMRs (Table S10). Our most significant DMR in PFC astrocytes is located in the 3ʹ UTR of the *DLX1* gene (Sidak *p =* 2.75 × 10^−5^), previously associated with neuronal tau accumulation in progressive supranuclear palsy.^55^ Notably, we also observed an astrocyte‐associated DMR on the *HOXC4* gene, containing five probes (Sidak *p =* 0.024), uncovering potential additional roles for the HOX clusters in AD pathology. DMR analysis of the temporal cortex showed 17, 19, 16, and 19 significant DMRs associated with Braak stage in astrocytes, oligodendrocytes, neurons, and endothelial cells, respectively (Table S11). In the oligodendrocyte pool, we noted a DMR on a different HOX gene, *HOXC8* (Sidak *p =* 2.52 × 10^−5^), as well as multiple DMRs on a zinc‐finger protein, *ZIC4*. We observed no significant cell‐type‐specific DMRs within some previously identified popular Braak stage‐associated genes, including those within the HOXA clusters and ANK1, but we did identify one on the *CDH1* gene in astrocytes (Sidak *p =* 0.014) and another on the *KCNQ1* in neurons (Sidak *p =* 0.001), genes previously identified as housing DMPs in association with Braak stage.^5^

Cell‐type deconvolution limits the power of brain region‐wide association testing because of the inherent dilution of signals from aggregated tissues to estimated cell types. In a previous meta‐analysis of bulk cortical tissues (including PFC and TC), which utilized some of the same cohorts as those used in this study,^14^ Smith and colleagues generated DMP‐level information in association with Braak stages. We leveraged these data to ask which cell type contributed to the “bulk” Braak signal by performing a rank‐based enrichment analysis of effect‐size distributions across both cortical regions for each cell type, using region‐specific brain Smith‐DMPs. Relative to the overall distribution of effect sizes for each cell type, within the PFC, rank‐based analysis shows that both astrocytes and neurons exhibit a significant shift toward larger ESs of Smith‐DMPs (Figure 4B, Table S12), with a median absolute z‐score shift of 0.32 and 0.47, respectively (Wilcoxon *p =* 6.49 × 10^−9^ and 9.10 × 10^−16^ respectively), and AUC of 0.61 and 0.65, respectively (perm‐*p <* 0.001 for both). Oligodendrocytes and endothelial cells do not show a significant ES enrichment for Smith‐CpGs in the PFC. In the TC (Figure 4C), the effect size distribution of Smith‐CpGs was not enriched in any one cell type. The neuronal and astrocytic ES enrichment for Smith‐CpGs within the PFC is also evident in the DMR analysis, where neurons shared at least three regions (Sidak *p <* 0.05) within genes implicated by Smith et al., including a highly significant DMR at the *GPR37L1* gene (Sidak *p =* 2.15 × 10^−5^) (Figure 4D), and astrocytes shared one DMR at *RUNX1T1* (Sidak *p =* 0.019) (Figure S6). To further delineate the potential contributions of these two cell types to Braak stage, we examined how consistently the cell‐type effects conformed in direction with the bulk tissue estimates from the Smith et al. study through a series of rank‐based comparisons, starting with Smith‐CpGs with the largest estimated effects. Leading‐edge concordance analysis showed both neurons and astrocytes exhibited high directional concordance to Smith‐CpGs across the ranked distribution (Figure 4E). Notably, among the strongest Braak‐associated CpGs (highest ES), astrocytes showed a higher early concordance (Δ = 0.05 between astrocyte and neuronal concordance across the top 50 ranked Smith‐CpGs), whereas neuronal concordance increased gradually and slightly exceeded astrocytes when the broader set of Smith‐CpGs was considered. Unlike our age‐ and AD‐association analyses, which are summarized by DMP counts, our Braak‐stage findings for astrocytes and neurons are additionally supported by count‐independent rank‐based ES enrichment analysis, which is not subject to the same abundance‐related confound and therefore provides converging, orthogonal evidence for the specific involvement of these two cell types.

### 3.4 AD‐dependent effects on age‐associated DMPs vary by cell type

**FIGURE 5:**
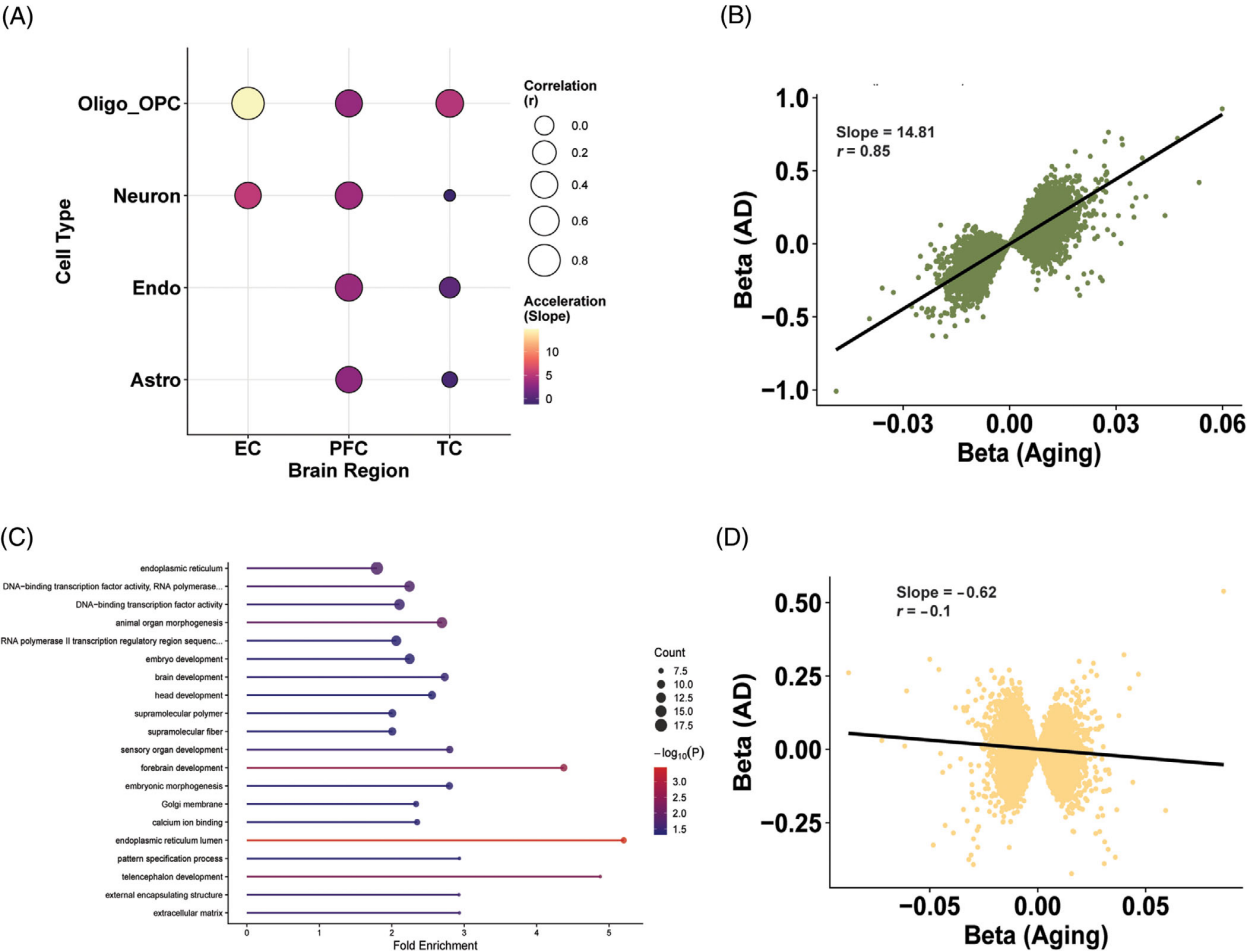
AD‐dependent effects on age‐associated DMPs vary by cell type. (A) Bubble plot showing differential cell‐type aging burden associated with AD status across all cell types × cortical regions analyzed. The color scale represents the slope of regression, indicative of an increase in the magnitude of ES in AD (i.e., acceleration). Bubble size indicates correlation of effect sizes at age‐associated DMPs (*p <* 0.05) between age and AD associations. (B) Correlation scatter plot of effect sizes at age‐associated DMPs between age and AD associations in EC oligodendrocytes (*r =* 0.87, slope = 0.64). (C) Gene Ontology enrichment analysis for directionally concordant age‐AD CpGs at nominal significance in EC oligodendrocytes, ordered by counts. The *x*‐axis represents fold enrichment. Color scale represents −log_10_(*p*). (D) Correlation scatter plot of effect sizes at age‐associated DMPs between age and AD associations in TC astrocytes (*r =* −0.05, slope = −0.02).

Epigenetic age acceleration is a known feature of AD. One recent study showed this age acceleration is driven by the overall glial population in the temporal lobe of AD patients,^56^ suggesting the existence of age‐related cell‐type vulnerability patterns associated with disease. Therefore, we wanted to assess whether AD‐based methylation remodeling conforms with, amplifies, or diverges from normal age‐associated methylation programs in a cell‐type‐specific manner. We focused on cell‐ and brain region‐specific age‐associated CpGs (identified in our age‐association analysis) and tested whether AD‐associated methylation effects (ESs) at these sites were concordant in direction (correlation) and magnitude (regression slope) compared to ESs associated with normal aging. Strikingly, we observed region‐dependent variability at age‐associated sites (nominal) in AD (Figure 5A). For example, while EC and TC exhibited cell‐type‐specific patterns, most cell types across the PFC showed low to moderate correlation of ESs between age and AD, as well as minimal ES amplification with AD.

For age‐associated DMPs across all brain regions, the oligodendrocyte fraction exhibited moderate to high correlation (*r =* 0.4 to 0.85) between ES in age and AD and showed an increased ES magnitude with AD. The most extreme age‐to‐disease ES amplification was seen in EC oligodendrocytes (Figure 5B) (slope = 14.81, *r =* 0.85, *p <* 2.2 × 10^−308^), consistent with our previous observations of shared age‐AD DMPs (Figure 3E), suggesting that disease‐associated changes extend pre‐existing age‐related patterns. Of the age‐associated DMPs that exhibit concordance in ES direction with AD in the EC oligodendrocytes, GO analysis shows an enrichment for broad neural development‐related processes (Figure 5C), suggesting an overall extension of developmental programming pathways with AD.

Interestingly, in TC, all cell types other than oligodendrocytes showed much lower amplification of ES with AD and exhibited poor correlations between age and AD ES. For example, both astrocytes and neurons show near‐zero correlation between age and AD ES (*r =* −0.1 for both, *p <* 2.42 × 10^−46^), with astrocytes exhibiting low age‐to‐disease ES acceleration (Figure 5D, slope = −0.62), suggesting that AD‐associated methylation remodeling in these cells is not a simple extension of age‐associated epigenetic programs. Tong et al. highlighted a glial signature in the temporal lobe in their cell‐type epigenetic age study.^56^ We now show that this signature is likely dominated by oligodendrocytes, but not astrocytes or other cell types, revealing clear subglial effects in disease states.

### 3.5 Cell‐type‐resolved methylation findings converge with independent epigenomic and transcriptomic studies of AD

Our cell‐type‐resolved methylation findings are also consistent with, and extend, a rapidly growing body of single‐cell and single‐nucleus chromatin accessibility and transcriptomic studies of AD. Single‐nucleus multi‐omic profiling of the PFC identified an oligodendrocyte‐associated cis‐regulatory module linked to *APOE* and *CLU* in AD,^57^ while cell‐type‐resolved H3K27ac profiling found that oligodendrocyte‐enriched glia harbored the majority of amyloid‐associated differential histone acetylation, concentrated near AD risk loci and myelin‐related genes.^58^ A large‐scale, multi‐region, single‐cell transcriptomic atlas similarly identified glial‐enriched gene modules tied to cholesterol biosynthesis and lipid metabolism,^59^ and single‐nucleus multiomic profiling spanning PFC, EC, and HC implicated astrocyte‐specific transcription factors in disease‐associated gene regulation.^60^ These findings converge with our own identification of oligodendrocytes as the predominant AD‐associated cell type in the EC, including our novel *ABCA1* DMP within the APOE‐lipidation pathway. Similarly, a recent single‐nucleus multi‐omic preprint of Down syndrome‐associated AD identified PFC astrocytes as a selectively vulnerable cell state marked by coordinated transcriptional and chromatin‐accessibility reprogramming,^61^ while another study identified disease‐associated chromatin accessibility profiles to be linked to astrocytes in primary tauopathies^62^ – a striking parallel to our identification of PFC astrocytes as the predominant cell type associated with both age and Braak‐stage methylation in this region. Together, this cross‐modality convergence lends independent support to the cell‐type‐specific patterns identified here through DNA methylation.

## 4 DISCUSSION

In this study, we performed a comprehensive analysis of cell‐type‐resolved methylomic changes associated with healthy aging, AD status, and Braak stage across several brain regions. We utilized 13 previously published AD‐EWAS cohorts and integrated region‐stratified meta‐analysis, with rank‐based ES enrichment and DMR discovery to uncover novel, distinct roles for glial cells in these processes. We refined prevailing methylation models of AD and demonstrated distinct age‐ and disease‐associated programs that fundamentally differed in scale and coherence, depending on cellular origin and anatomical context.

Across all analyses, we observed prominent DNA methylation changes associated with age. Cell‐specific effects were highly region‐specific, represented by higher DMP burden in glial cells (astrocytes and oligodendrocytes) within the cortex and neuronal cells in the cerebellum. We identified 669 DMPs and 224 DMRs in PFC astrocytes associated with age. Several of these mapped to genes involved in homeostatic astrocyte function, including a highly significant DMR within *ATP1A1*, involved in ion transport and synaptic communication. We also identified several CpG sites that were differentially methylated within the *VPS52* gene, one of which was common to a previous age EWAS of sorted brain cell types.^5^ *VPS52* is a core component of the Golgi apparatus and was previously implicated in association with *LRRK2* in Parkinson's disease astrocytes.^63^ Our data now point to a novel potential role for DNA hypermethylation at sites within this gene in astrocytes. To some extent, PFC astrocytes also exhibit shared aging trajectories with other brain regions (e.g., TC), suggesting that glia undergo profound epigenetic remodeling with age in the cortex, reflecting changes in CNS homeostasis, metabolism, and neuronal support processes.^64^, ^65^

In contrast, region‐stratified analyses of AD status revealed far fewer significant associations, with oligodendrocytes exhibiting the highest number of AD‐associated DMPs in specific cortical regions. Notably, within the EC, we identified a novel hypermethylated DMP within the *ABCA1* gene, which is involved in cholesterol and lipid homeostasis and is a key partner of the AD‐risk factor, *APOE*.^66^, ^67^ Dysregulation of overall *APOE* lipidation status^68^, ^69^ and accumulation of lipid droplets due to inefficient fatty acid and cholesterol metabolism in oligodendrocytes was previously linked with AD‐relevant impaired myelin repair, white matter damage, and neuroinflammation.^70^, ^71^, ^72^ Astrocytes showed a moderate AD signal in the PFC, particularly in genes involved in calcium signaling and those encoding ion channels. Neurons, however, exhibited relatively few cortical DMPs, indicating that a greater proportion of significant AD‐associated methylation changes detected in the cortex occur in glial cell types.

Despite its central role as a pathological index for AD, Braak stage produced only a handful of DMPs that survived multiple testing corrections. This absence of strong Braak‐associated DMPs may also be influenced by the inherent nature of distributed tau pathology across the cortex and cell‐type resolution artifacts. In fact, Braak stage is likely highly dependent on both spatial and temporal patterning across cell types, and, with modest methylation changes, these properties challenge single‐CpG significance testing. To address this limitation, we employed a rank‐based enrichment approach to ask whether CpG sites previously identified as significantly associated with Braak stage in bulk cortical tissues^14^ show preferential enrichment in their ES distribution in distinct cell types. Using rank‐based analysis, AUC‐based enrichment, and leading‐edge concordance metrics, we identified clear, cell‐type‐specific patterns that were obscured in FDR‐based single‐CpG testing. Both astrocytes and neurons in the PFC showed significant effect‐size enrichment of Smith‐CpGs relative to their respective overall ES distributions across all tested CpGs, whereas no individual cell type had a pronounced effect in the TC. Astrocytes and neurons in the PFC also differed in how they were enriched for Smith‐CpGs. Directional concordance analysis revealed a somewhat rank‐dependent division of labor – astrocytes displayed near‐complete directional agreement with the top 20% of Braak‐associated CpGs that marked advanced pathology (as evidenced by higher ES), whereas neuronal concordance became greater when a broader set of Braak‐associated CpGs was considered. This model suggests a scenario where astrocytes align more strongly with the very highest‐effect Braak‐CpGs, likely playing a dominant role in responding to severe pathology and exhibiting functional changes in relation to energy metabolism, homeostasis, and neuroinflammatory signaling axes.^65^, ^73^, ^74^ Neuronal involvement, in contrast, aligns with broader changes underlying gradual pathological remodeling across disease stages.

One key unresolved question in AD epigenomics is whether disease‐associated changes reflect a mere acceleration of normative aging or a distinct disease‐relevant epigenetic remodeling. We find that age‐associated methylation trajectories are propagated in a spatial, lineage‐dependent manner. Oligodendrocytes show the strongest ES correlation of age‐associated DMPs in AD, with trajectory analysis indicating an increase in magnitude of these processes across all cortical regions. Astrocytes, in contrast, show region‐specific diversification – within the TC, age‐associated ES estimates were largely not correlated with disease state, suggesting that AD‐related epigenetic remodeling in TC astrocytes exhibits a divergence from aging‐related methylomic patterns. These results support a model of lineage‐defined trajectories, where oligodendrocyte aging programs define a regulatory axis along which disease progression preferentially occurs, but other cells, including astrocytes, exhibit a decoupling of age‐related programs in lieu of disease‐specific epigenetic remodeling.

There are some limitations to our study. We resolved cell‐specific methylation profiles from bulk tissue using a deconvolution paradigm.^20^, ^42^, ^75^ While this provides novel insights into cell‐level dynamics in aging and AD‐relevant processes, low abundance of some cell types (e.g., microglia), regional and disease‐specific cell composition shifts, tissue‐level heterogeneity, and accuracy of methylation‐based cell‐type reference panels may obscure findings. Neurons also exhibit higher levels of DNA hydroxymethylation compared to other cells. Since traditional bisulfite treatment does not distinguish between methylated and hydroxymethylated cytosines, a composite signal arising from both methylation states may conceal key findings in neurons. Our study focused on identifying glial‐specific epigenetic programs, but epigenetic patterning in endothelial cells may provide further insight into brain aging and AD pathology.

Our cell‐type deconvolution analysis was limited in power, despite having many samples. Such models incur high multiple testing penalties that substantially reduce the effective sample size per test. This may partly explain why we did not replicate some known AD‐associated DMPs (e.g., HOXA clusters), although it is possible that effects may be dispersed across cell types, which may not be well captured by our cell‐type‐specific interaction models. Moreover, cell‐type‐specific inferences also depend on phenotype balance and the abundance and variability of estimated cell‐type fractions. For instance, low‐abundance or minimally variable cell types are likely to yield less stable estimates, whereas greater variability in estimated fractions should provide greater statistical power to detect cell‐type‐specific associations – which is important to consider when interpreting the observed differences in DMP counts across cell types. Although meta‐analysis improves power, several of our individual cohorts are relatively modest in size, and despite using cohort‐level QC, inflation correction, and heterogeneity assessment to reduce unstable estimates, these analyses remain underpowered relative to the scale required for definitive cell‐type‐specific methylation discovery. Further, most cohorts considered for this study provide only binary disease status or Braak stage data. AD pathology is multivariate – future studies utilizing other quantitative measures (e.g., amyloid pathology burden, amyloid‐tau neurodegeneration [ATN], or Consortium to Establish a Registry for Alzheimer's Disease scores) may generate stronger cell‐type‐specific association signals. We did not perform sex‐stratified or sex‐by‐phenotype interaction analyses, which would have required substantially larger sample sizes per cohort, region, and cell type. Also, uneven coverage of relevant brain regions in available array‐based datasets is another limitation.

Although we characterized cell‐specific age‐to‐disease trajectories, since most EWAS data were from AD‐related cohorts, the age range of our neurologically healthy controls was relatively limited. Assessment of normative aging processes in the decades immediately preceding AD might inform whether methylation patterns could be predictive of disease onset. Finally, while we adjusted for age in all AD models, age and AD diagnosis remain strongly correlated at the population level, and covariate adjustment alone cannot fully remove the effects of this underlying correlation. Our comparison of age‐ and AD‐associated ES was therefore intended, in part, to distinguish shared signals from AD‐specific signals, although complete separation of aging and late‐onset AD pathology remains an unresolved challenge. Ultimately, while we found several relevant, novel DMP loci that implicate glial cells in aging and AD, follow‐up large‐scale validation studies that utilize whole‐epigenome, sorted‐cell populations, and single‐cell methylomic assays and/or in vitro models, balanced for better representation of sex‐ and disease‐relevant regions that could not be assessed in this study, are warranted.

In summary, we provide a refined view of epigenetic regulatory states in AD by revealing distinct, lineage‐specific methylomic programs underlying brain aging and disease. Using a computational deconvolution approach, we show astrocyte‐ and oligodendrocyte‐dominated signatures in the aging cortex, oligodendrocyte‐dominated AD associations in the EC, and astrocytes and neurons as key contributors to Braak‐associated epigenetic remodeling in the PFC, where astrocytes show more directional concordance at loci highly associated with Braak stage and neurons exhibit a broader concordance with these disease‐associated CpGs. We also uncovered a distinct age‐to‐disease trajectory, where oligodendrocytes exhibit an age‐to‐disease amplification of ES across cortical regions, impacting developmental programs, but astrocytes show a divergence from age‐associated epigenetic programs with AD. These insights extend tissue‐level findings and underscore the importance of cell‐type‐resolved, rank‐aware approaches for dissecting the epigenomic landscape of AD.

## AUTHOR CONTRIBUTIONS

Uchit Bhaskar undertook data analysis and bioinformatics. Uchit Bhaskar, Mark Z. Kos, and Melanie A. Carless conceived the idea and developed the project. Melanie A. Carless provided funding support. Uchit Bhaskar, Mark Z. Kos, and Melanie A. Carless drafted the manuscript. All authors read and approved the final submission.

## CONFLICT OF INTEREST STATEMENT

The authors declare no conflicts of interest. Author disclosures are available in the Supporting Information.

## CONSENT STATEMENT

We thank the authors of all prior publications that made their data publicly available, making this analysis possible, as well as the donors and families who made this research possible.

## Supporting information



Supporting Information

Supporting Information

Supporting Information

## Data Availability

Data used in this study were obtained from publicly available sources and were deidentified. See https://github.com/u-bhaskar/Cell-Meta-Analysis-Bhaskar.git.
