# Supplementary material for: A cell‐type‐resolved meta‐analysis reveals glial DNA methylation changes associated with aging and Alzheimer's disease
Source: Alzheimers Dement. 2026 Sep 24;22(9):e71864. doi: 10.1002/alz.71864 (PMC13602172; doi:10.1002/alz.71864)
Supplement: Supplementary file 2 [file ALZ-22-e71864-s003.docx]

**SUPPLEMENTARY FIGURES**

**Figure S1.** Mean and variability of cell-type proportions inferred from DNA methylation deconvolution show no correlation with DMP burden. Brain regions are represented by different colors, and cell types are represented by different shapes. Spearman’s rank correlation coefficient (ρ) was calculated for each analysis.

a) Representative correlation scatter plot of mean estimated cell proportion and number of significant age-associated DMPs, across four brain regions.
b) Representative correlation scatter plot of the SD of estimated cell proportion and number of significant age-associated DMPs, across four brain regions.
c) Representative correlation scatter plot of mean estimated cell proportion and number of significant AD-associated DMPs, across four brain regions.
d) Representative correlation scatter plot of the SD of estimated cell proportion and number of significant AD-associated DMPs, across four brain regions.

**Figure S2.** Bubble plot showing differential methylation burden associated with age across all cell types x regions analyzed, at a Bonferroni-adjusted significance of 0.05. Color scale represents proportion of sites seen to be hypermethylated (teal) and hypomethylated (orange). Size of bubbles indicates number of significant CpGs.

**Figure S3. a)** Manhattan plot for the meta-analysis of age-associated DMPs in entorhinal cortex oligodendrocytes, with the 20 most significant DMPs annotated with either CpG ID or Illumina UCSC gene name or both. X-axis shows chromosomes 1-22 and y-axis shows -log_10_(p), with the horizontal red line denoting FDR significance (0.05). **b)** Lollipop plot of the 15 most enriched GO terms for age-associated DMPs in EC oligodendrocytes, ordered by counts. X-axis represents fold-enrichment and color scale represents -log_10_(p).

**Figure S4. a)** Venn diagram of significant age-associated CpGs in PFC astrocytes versus nominal hits in TC astrocytes. **b)** Correlation plot of effect sizes at 65 common CpG sites between the PFC and TC astrocytes (r: Pearson’s correlation coefficient).

**Figure S5. a)** Bubble plot showing differential methylation burden associated with AD status across all cell types x regions analyzed, at a Bonferroni-adjusted significance of 0.05. Color scale represents the proportion of sites seen to be hypermethylated (teal) and hypomethylated (orange). Size of bubbles indicates number of significant CpGs.

**b)** Bar plot of the number of significant DMPs (FDR < 0.05) by cell type in EC, in association with AD disease status.

**Figure S6.** Differentially methylated region (DMR) analysis identifies regions previously identified as Braak-associated DMPs. Representative genome visualization plot of one such DMR in astrocytes at the *RUNX1T1* locus. The red track shows p-value significance, and the blue track shows ES of CpGs at this DMR in the meta-analysis. The orange track shows the DMR, and the genome tracks are shown below.
